# Translation and Validation of American Diabetes Association Diabetes Risk Test: The Malay Version

**DOI:** 10.21315/mjms2022.29.1.11

**Published:** 2022-02-23

**Authors:** Nurul Fatihah Mohd Fauzi, Sharifah Wajihah Wafa, Abdullah Mohd Ibrahim, Naresh Bhaskar Raj, Mat Hassan Nurulhuda

**Affiliations:** 1 School of Nutrition and Dietetics, Faculty of Health Sciences, Universiti Sultan Zainal Abidin, Terengganu, Malaysia; 2 School of Rehabilitation Science, Faculty of Health Sciences, Universiti Sultan Zainal Abidin, Terengganu, Malaysia; 3 Faculty of Medicine, Universiti Sultan Zainal Abidin, Terengganu, Malaysia

**Keywords:** ***Keywords:*** American Diabetes Association, diabetes risk test, pre-diabetes, screening, type 2 diabetes mellitus

## Abstract

**Background:**

Early detection of high-risk people for type 2 diabetes mellitus (T2DM) using a simple, non-invasive and cost-effective assessment tool helps to identify and prevent members of the community from developing this disease. Therefore, this study aims to translate the American Diabetes Association (ADA) diabetes risk test for Malaysians and then evaluate its validity.

**Methods:**

This cross-sectional study was conducted between March 2019 and April 2019. The instrument underwent forward and backward translation according to Behling and Law’s technique. Content validity was performed by two experts and face validity was conducted among 35 convenience samples from Kota Bharu, Kelantan. Both were analysed using content validity index and face validity index, respectively.

**Results:**

All respondents were Malay, and had attained tertiary education with a mean (standard deviation [SD]) age of 20.63 (2.80) years old and BMI of 30.45 (5.99). Among the respondents, 57.1%, 94.3% and 80% were female, single and having a household income below RM1,500, respectively. The Malay translated instrument achieved high I-content validity index (CVI) [0.5–1.0] and S-CVI/Ave [0.93] as well as high I-face validity index (FVI) [0.86–0.97] and S-FVI/Ave [0.91] for understandability, and high I-FVI [0.77–0.91] and S-FVI/Ave [0.85] for clarity.

**Conclusion:**

The Malay version of the ADA diabetes risk test was found to be a valid survey instrument to be used for the Malaysian adult population.

## Introduction

Diabetes prevalence has risen considerably over the past few decades in both developing and developed countries and has become a primary health concern worldwide (1). According to the 9th International Diabetes Federation (IDF) (2), about 463 million adults aged 20 years old–79 years old have diabetes and the number will continue to rise to 700 million by the year 2045. Furthermore, 79% of them live in low- and middle-income countries, with most countries having a growing number of people with type 2 diabetes mellitus (T2DM) and 374 million people are at increased risk for developing T2DM (2). In Malaysia, the prevalence of diabetes has increased to 18.3%, which means that around 3.9 million Malaysians or approximately 1 in 5 Malaysian adults have diabetes (3).

There are a range of screening tests available which include risk assessment questionnaires, biochemical tests and combinations of the two (4). The currently available biochemical tests are blood glucose or urine glucose measurements and blood HbA1c or blood fructosamine measurements (4). In Malaysia, as described in the Malaysia Clinical Practice Guideline (CPG) for diabetes mellitus, the current methods used to screen T2DM are capillary plasma glucose, venous plasma glucose and blood HbA1c. Even though these screening methods could detect individuals at high risk for diabetes or asymptomatic diabetes, they are considered as invasive, costly and time consuming (5). In addition, blood glucose concentration fluctuates easily and greatly over a 24-h period and from day to day, and can only provide information on an individual’s current glycaemic status (5–6). Therefore, there is a dire need for an assessment tool that can help medical professionals track the risk factors that can lead to diabetes easily, efficiently and non-invasively, using information collected during general practice visits such as a history of smoking, body mass index, sex, age, family history and the use of anti-hypertensive and steroidal medications (5, 7–9). Population-wide diabetes screening with a generic risk calculator is more appropriate than using invasive tests like HbA1c or blood glucose. Countries such as Finland, Canada and Thailand are regularly using standard risk calculators to assess who should be subjected to invasive T2DM diagnostic testing (5, 10–11).

The American Diabetes Association (ADA) diabetes risk test was developed as a screening tool to classify high-risk subjects in the community and to raise awareness of modifiable risk factors and healthy lifestyle (5). The ADA diabetes risk test scoring includes seven questions (total score of 0–11) regarding the age, gender, gestational diabetes mellitus (GDM), family history of diabetes, high blood pressure, physical activity and obesity (based on body mass index (BMI) via a weight-height chart). Those having scores of 5 and more are considered to be at high risk of having diabetes. Studies reported that the risk of diabetes increases with age (12–13). Diabetes is more likely to occur in people who are older, owing to concurrent increases in insulin resistance linked to obesity and inactivity (14). Previous studies also reported that men are more prone to develop diabetes than women (15–19), where men under 55 years old have a higher chance of developing heart disease and diabetes than women (20). Studies reported that women who were previously diagnosed with gestational diabetes during pregnancy have a higher chance of developing diabetes as well as cardiovascular diseases later in life compared to women who have normoglycemic pregnancy, with more than sevenfold increased risk of developing T2DM (21–22). According to several population-based studies, family history of diabetes is closely related to the development of diabetes (23). Being overweight or obese are the most significant diabetes predictors (24). Studies conducted in multiple countries have discovered a stronger relationship between anthropometric markers and the occurrence of T2DM (25–27). Notably, people with high blood pressure were discovered to have a 50% increased chance of developing T2DM (28).

The risk screening method for diabetes was originally developed from a risk prediction model using nationally representative data from the National Health and Nutrition Survey (NHANES) 1999–2004 and includes six easily answered health-related questions. Validation of this risk model was based on 2005–2006 data from the same source, in addition to the baseline data from two large cohorts, the Community Atherosclerosis Risk Study and the Cardiovascular Health Study. The risk questionnaire based on this predictive model reported 79% sensitivity, 67% specificity, 10% positive predictive value (PPV), 99% negative predictive value (NPV) and area under the receiver-operating characteristic curve (AUROC) of 0.83 (29). As a result of these positive outcomes, the ADA adopted it and added another question on the history of gestational diabetes in women (30). Based on the recent findings of Woo et al. (31) using the ADA diabetes risk test, the risk test continued to maintain its reasonably good accuracy with an AUROC of 0.725 when applied to a population of Chinese adults. The results also indicated that the ADA diabetes risk test had a strong validity in identifying Chinese adults with undiagnosed diabetes. Another study that evaluated the usefulness of the ADA diabetes risk test in predicting T2DM or pre-diabetes among the Indian population revealed that the ADA risk scoring was a significantly useful tool for identifying people who have pre-diabetes and T2DM in the population. The study reported that patients with a score below 5 had mean HbA1c of 4.7 ± 0.1, while patients with score of 5 or more had mean HbA1c of 6.07 ± 0.02 (32). The adaptation of the ADA diabetes risk test among the Indonesian adult population demonstrated significant results in screening hyperglycaemia with AUROC of 0.71 (95% Cl: 0.60, 0.81). In addition, the risk status reportedly had the greatest AUROC value when a cut-off of ≥ 5 is used with an overall accuracy, sensitivity and false negative rate (FNR) of 66%, 68% and 32.36%, respectively (33). From the results of these previous studies, it can be concluded that the ADA diabetes risk test might be a beneficial diabetes risk tool for the Asian population in detecting T2DM as well as pre-diabetes. However, evidence supporting the screening of adults for diabetes risk using a diabetes risk assessment tool in Malaysia is limited. In Malaysia, development or translation and validation of diabetes risk tools in the Malaysian perspective has been scarcely documented. The only available study that evaluated the use of diabetes risk assessment tools to predict T2DM in Malaysia was done by Oo et al. (34). The study modified the available Finnish T2DM risk assessment tool to identify individuals at risk of diabetes. The study reported that approximately 60% of the respondents had moderate to high risk of developing T2DM in the next 10 years. However, this study did not translate the questionnaire into the mother tongue of the respondents, which might pose a problem to certain people who do not understand English. This study also did not perform face validity assessment to assess the extent of the Malaysian people’s belief that the assessment items meet the targeted constructs as well as the assessment objectives (35).

By using available tools to forecast the risk of patients developing diabetes, physicians and other healthcare professionals can become more aggressive in promoting healthy lifestyle interventions that may reduce the risk of diabetes (7). The ADA diabetes risk test has been shown to be more effective in predicting people at high risk of developing diabetes compared to other diabetes risk assessment tools such as the Finnish Diabetes Risk Score (36). Furthermore, the ADA diabetes risk test has several other benefits including being simple and applicable in different community or clinical settings, can be calculated quickly and even manually, with no strict need for a calculator or a computer, and requiring minimal time (30). Therefore, the aims of the study were: i) to translate the ADA diabetes risk test into Malay language using Behling and Law’s technique (37) and ii) to examine the content and face validities of the Malay translated ADA Diabetes Risk.

## Methods

This cross-sectional study was conducted between March 2019 and April 2019. Two experts in the fields of nutrition and dietetics as well as 35 target users participated in this study.

### ADA Diabetes Risk Test

The ADA diabetes risk test is a risk score based on seven criteria that predict the risk of developing diabetes, including age, gender, family history of diabetes, history of gestational diabetes in women, history of hypertension, physical activity and BMI. Participants were required to answer yes or no to all the questions except for age and BMI. For age and BMI, the score ranged from 0 to 3. For age, 40 years old and below was scored as 0, 40–49 years old was scored as 1, 50–59 years old was scored as 2 and 60 years old or above was scored as 3. Meanwhile for BMI, normal and underweight were scored as 0, overweight as 1, obese class I as 2 and obese class II as 3. For gender, being a male was scored as 1 and female as 0. For physical activity level, a negative response was scored as 1 and a positive response was scored as 0. For the other parameters, negative response was scored as 0 and positive response was scored as 1. The total ADA diabetes risk test score was the sum of scores from all seven questions, and participants who scored five and above were considered as at high-risk of developing diabetes.

#### Stage 1: Malay Translation of ADA Diabetes Risk Test

To translate the original English ADA diabetes risk test into the Malay language, Behling and Law’s technique was used (37). This technique required three translators with excellent proficiency in both the target language and the original language. They were experts in the Malay language as well as in linguistic and literature. The first translator translated the English version of the ADA diabetes risk test into the Malay language. Then, the second translator back-translated the translated version into the original language, and the third translator compared the original and back-translated versions and edited them to obtain the matched Malay version. After minor adjustments, a final English version was used to re-evaluate the Malay version. The final Malay version of the instrument was ready to be used in the study after further discussion.

#### Stage 2: Content and Face Validations of Malay Version of ADA Diabetes Risk Test

The validation of the Malay version of the ADA diabetes risk test was conducted in two steps, which are content validation and face validation. Content validation aimed to assess the relevance of all seven ADA diabetes risk test items and was conducted on two experts in the fields of nutrition and dietetics who were also experts in diabetes and clinical nutrition research. This is the minimum acceptable number of experts required for content validity as stated by Davis (38). They were required to evaluate each item using a four-point Likert scale, whereby 1 indicated that the item was not relevant, 2 indicated that the item was somewhat relevant, 3 indicated that the item was relevant and 4 indicated that the item was highly relevant. Extra columns were provided for the experts to leave their comments in. For the content validity index (CVI), scores of 3 and 4 were recategorised as 1 (relevant) and scores of 1 and 2 as 0 (not relevant). CVI was determined by calculating the scale average using the formula S-CVI/Ave = sum of I-CVI scores/number of item, where I-CVI was calculated based on the formula I-CVI = agreed item/number of expert (38–41).

**Figure 1:**
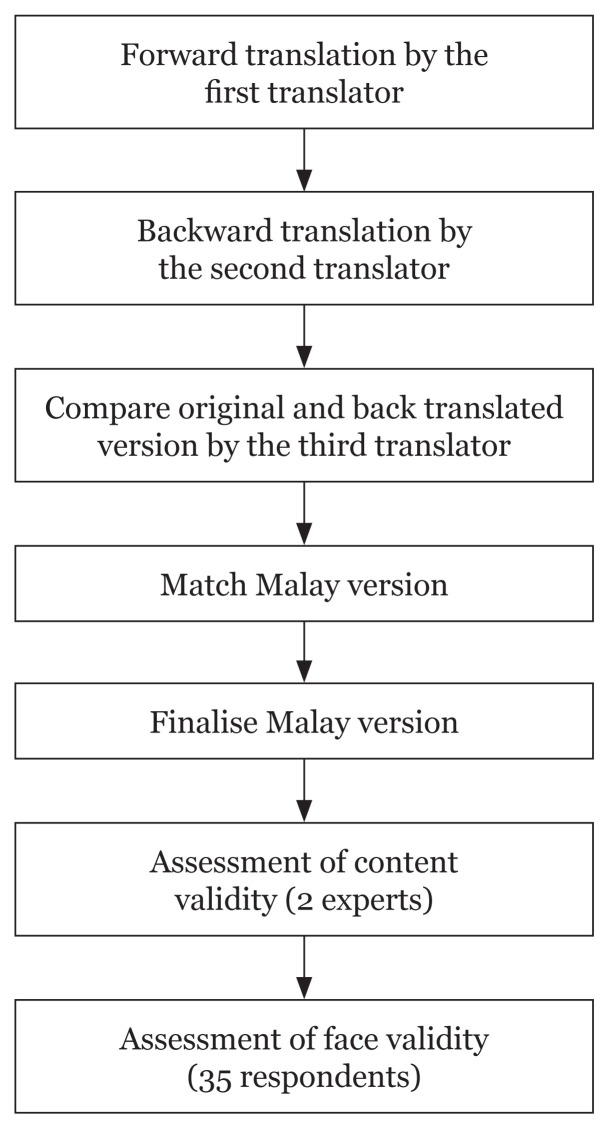
Flow chart of the validation process

Face validation was conducted on a convenience sample of 35 target participants living in Kota Bharu, Kelantan, that fulfilled the inclusion criteria such as non-diabetic adults aged between 18 years old and 65 years old, as well as being able to read and write in Malay language. Those with cognitive impairment, illiteracy in Malay, as well as problems listening and understanding the Malay language were excluded. A cohort of 35 respondents were chosen, which yielded a respondent-to-item ratio of 5:1 in line with the suggestion by Gorusch (42). Kota Bharu, Kelantan, was selected as the study location because Kelantan ranked second and third in terms of the prevalence of pre-diabetes and undiagnosed diabetes, respectively (43). Additionally, according to the Household Income and Basic Amenities Survey Report 2019 by the Department of Statistics Malaysia (44), Kota Bharu was reported as the most populous district in Kelantan. The respondents were asked to rate the Malay version of the ADA diabetes risk test with regards to the understandability and clarity of the translated items using a four-point Likert scale ranging from ‘item not understandable/not clear’ to ‘item very understandable/very clear’. A rating of 1 indicated that the item was not understandable/not clear, 2 indicated that the item was somewhat understandable/somewhat clear, 3 indicated that the item was understandable/clear and 4 indicated that the item was very understandable/very clear. For the face validity index (FVI), the score from 35 participants was recategorised as 1 for scores of 3 and 4 (understandable and clear) and as 0 for scores of 1 and 2 (not understandable and not clear). FVI was determined by calculating the scale average using the formula S-FVI/Ave = sum of I-FVI scores/number of item where I-FVI was calculated based on the formula I-FVI = agreed item/number of respondent (45). Figure 1 illustrates an overview of the validation process.

## Results

### Stage 1: Translation

In the translation of the ADA diabetes risk test questionnaire, the translators suggested that sentences be modified wherever possible, while at the same time retaining the meaning of the original version. Direct translation is not commonly used for all questions. For instance, the sentence ‘write your score in the box’ when translated into Malay would be ‘tulis markah anda di dalam kotak’. The translators suggested modifying the word ‘tulis’ into ‘isi’ which was more appropriate and added the word ‘below’ which means ‘di bawah’ in Malay to make the sentence clearer. Meanwhile, the sentence ‘add up your score’ literally means ‘tambah markah anda’. The panels proposed to amend it into ‘jumlah markah’ since they proclaimed that the former meaning was more appropriate to be used in a full sentence such as while giving the instruction on how to total up the points.

Medical words such as gestational diabetes and diabetes were translated into ‘kencing manis ketika mengandung’ and ‘kencing manis’, respectively. In question 4, the word sister or brother was translated into ‘adik beradik’. For question 7, the units of measurement for height and weight were changed into centimetre (cm) and kilogram (kg), since both units are commonly used in Malaysia to measure height and weight. In addition, the weight range in the chart was also modified based on the World Health Organization (WHO) BMI cut-offs for Asian and Pacific populations (46).

For the sentence ‘your weight is less than the 1 point column (0 point)’, when directly translated it carries the meaning ‘berat badan anda kurang daripada lajur 1 markah’ which was quite confusing to understand. Therefore, the panels suggested adding the word ‘weight’ which means ‘berat’ to make the sentence clearer. Therefore, it was amended into ‘berat badan anda kurang daripada berat pada lajur 1 markah (0 markah)’. For the description of the test result, the sentence ‘a condition that precedes type 2 diabetes in which blood glucose levels are higher than normal’ which literally means ‘suatu keadaan yang mendahului/datang sebelum kencing manis jenis 2 di mana tahap gula dalam darah lebih tinggi daripada biasa’ was also quite confusing to understand. Therefore, the sentence was excluded and amended into ‘prediabetes is a condition in which blood glucose levels are higher than normal but not high enough to be classified as diabetes’ which means ‘pradiabetes merupakan suatu keadaan di mana tahap gula dalam darah lebih tinggi daripada biasa tetapi tidak terlalu tinggi untuk diklasifikasikan sebagai kencing manis’ in Malay language. The phrase is the definition of prediabetes. Paragraphs 2 and 3 in the description of the test result were excluded because they are not quite related for Malaysia. The linguistic issues discussed during the translation process are summarised in Table 1.

### Stage 2: Validation

#### Content Validation by Experts

No major corrections were needed for the content validation of the ADA diabetes risk test Malay version. The average CVI calculated was 93% or 0.93, which was clearly above the 0.80 cut-off score for two experts as in Table 2 (38). The experts evaluated each item’s relevance and one expert commented on the items number 2, 5 and 6. Only one item was amended as per the expert’s suggestion to make it clearer, which is item number 6 regarding the question about being physically active. Therefore, ‘get ≥ 150 min of moderate to vigorous intensity physical activity per week’ which when translated into Malay means ‘mendapatkan ≥ 150 min aktiviti fizikal intensiti sederhana hingga berat setiap minggu’ was added to the question. This is the recommended level for physical activity in adults (47). Items 2 and 5, which were about gender and history of hypertension respectively, were not modified since they are the conventional risk factors for diabetes that do not require measurements or invasive tests (31).

#### Face Validation by Target User

A total of 35 target respondents participated in this study consisting of 42.9% males and 57.1% females. The mean (SD) of age and BMI of all respondents were 20.63 (SD 2.80) years old and 30.45 (SD 5.99), respectively. All the respondents were Malay, where 94.3% were single and the rest were married. All of them also had attained tertiary level of education as their highest level of education. However, the majority of them (80%) have household income below RM1,500 (Table 3). The average FVI for the understandability of the Malay ADA diabetes risk test was calculated to be 0.91 (Table 4) and the average FVI for the clarity of the translated instrument was calculated to be 0.85 (Table 5). An average FVI of above 0.83 for both criteria (understandability and clarity) indicated that all items in the questionnaire were understandable and clear for the target participants (38, 40).

## Discussion

Early identification of people at increased risk of developing T2DM is important. The screening of populations to identify people at risk using self-assessment questionnaires is a common method to inspire changes in lifestyle. Diabetes screening through an evaluation of risk factors such as the diabetes risk test of ADA is recommended to guide healthcare providers on whether a diagnostic test such as blood glucose or HbA1c measurement is necessary or not.

The screening parameters are largely based on age and BMI as predictive factors, and these two risk factors also contribute significantly to the risk test scores. Obesity and diabetes are closely linked, with about 80% of diabetics being obese. Overweight and obese people are more prone to develop T2DM, especially if they have excess weight around their abdomen. Abdominal fat causes fat cells to release ‘pro-inflammatory’ chemicals, which can make the body less sensitive to the insulin it produces by interfering with the function of insulin responsive cells and their ability to respond to insulin. This is referred to as insulin resistance (48). Incidence of diabetes increases with age (8), where before the age of 30 years old, the incidence of T2DM is low in most populations, but it rises rapidly and continuously as people get older (49–51). The screening criteria recommend that even if there are no other risk factors present, regular diabetes testing should begin at age 45 years old and repeated every three years if the previous test result is negative (30). Other health-related questions raised by the risk test also include sex, family history of diabetes, history of gestational diabetes in women, as well as history of hypertension and physical inactivity, which are customary risk factors for diabetes without the use of measurements or intrusive testing. Recently, male sex has been considered a risk factor to developing T2DM (15, 17, 52–55). This might be due to central obesity which is associated with higher risk of T2DM in men, as men are more susceptible to android adiposity whereas women are more likely to have gynoid adiposity (56). Those with at least two first-degree (mother, father, brothers or sisters) biological relatives of the same bloodline with diabetes, at least one first-degree and two second-degree (maternal and paternal aunts, uncles, or grandparents) biological relatives of the same bloodline with diabetes or at least three second-degree relatives of the same bloodline with diabetes are at an increased risk of developing diabetes (23, 57). Women with gestational diabetes have a higher risk of developing T2DM. The risk of developing T2DM rises dramatically within the first 5 years after delivery, then levels off. At five years, the likelihood of developing T2DM is estimated to be 40%–50% (58). Hypertension is one of the components of metabolic syndrome (59) and metabolic syndrome can increase the risk of developing T2DM by 2-fold to 3-fold (60). Physical inactivity, defined as insufficient physical activity to meet current global recommendations by the WHO 2010 (61), is estimated to be responsible for 7% of the global burden of T2DM (62). For instance, sedentary behaviours, such as excessive screen time, are a risk factor for T2DM. Higher television viewing time was linked to an increased risk of T2DM in a meta-analysis of four prospective cohort studies (63). The advantages of using this risk test are its practicality and applicability to different community or clinical settings and the result can be rapidly calculated even by hand. The time required to complete the questionnaire is minimal and the use of a calculator or computer is not necessary (31).

The aim of this Malay version of the ADA diabetes risk test was to serve as a simple and easy diabetes risk calculator for adults in Malaysia that could be used in the primary care setting and by individuals themselves. To generate an equivalent questionnaire, literal translation is not enough. The questionnaire must be well translated linguistically and must be adapted to cultural differences to retain the validity of the content (64). On the other hand, the validation process attempts to ensure that the translated instrument has the same equivalent properties to measure the construct as the original version. Validity is defined as the degree to which an instrument measures what should be measured by the instrument (65–66).

The Malay version of the ADA diabetes risk test was found to be a valid survey instrument for Malaysian adults to evaluate the risk of diabetes. Even though the study was conducted in a single location (Kota Bharu, Kelantan) and involved only Malay respondents, the translated questionnaire might be a valid questionnaire to be used in other geographical areas as well as on other ethnicities in Malaysia for detecting the risk of developing T2DM, since most of the items in the ADA diabetes risk test are the criteria for testing T2DM and pre-diabetes in asymptomatic adults (i.e. women with history of gestational diabetes, BMI, family history of T2DM, hypertension and physical inactivity) as stated by Malaysia CPG for diabetes mellitus. The study findings revealed that the questionnaire had excellent content and face validities. The outcomes of the study will enable healthcare professionals to use the instrument to easily predict the risk of diabetes, since the assessment requires only personal medical information and simple non-invasive measures that should be appropriate for both health care providers and people with varying levels of education (31).

The instrument’s content validity was calculated based on the CVI values as it applied to the degree of consensus between the panellists (41). Based on the recommendation of the previous study, the level of agreement between two panellists is at 0.80 (38).

Face validity assessment evaluated the understandability as well as the clarity of the items to assess the risk of developing diabetes from the target participants’ point of view. The high overall FVI score for understandability (0.91) and clarity (0.85) showed that the original ADA diabetes risk test has been translated into understandable and clear sentences to all 35 targeted participants. Achieving a satisfactory level of face validity is an important criterion for a good questionnaire, as this will allow us to derive valid answers from the respondents.

The strength of this study is that the results obtained can be a benchmark for future iterations of the validation of the questionnaire. Despite its strength, this study also has its limitations. Studies with small sample sizes are common and are conducted for a variety of practical reasons such as time, budget, as well as other resource constraints (67). In this present study, we did not perform convergent validity between the Malay version of the ADA diabetes risk test total score and blood glucose or HbA1c to further test its validity in detecting diabetes. Furthermore, we also did not perform test-retest reliability to further test whether the answers given by the respondents were consistent over time. Another limitation was that this study was conducted in a single location (Kota Bharu, Kelantan) with a single population type (Malay) and may produce results that do not generalise to another location or population type (67). It is therefore recommended that further multicentre studies on this topic be conducted in the future, together with convergent and test-retest reliability studies, to further validate the translated questionnaire among the Malaysian population.

## Conclusion

This study has demonstrated that the Malay version of the ADA diabetes risk test is a statistically valid diabetes risk assessment tool for detecting diabetes risk among the adult population in Malaysia. We translated and validated the tool to predict diabetes risk. We hope that these preliminary results will enable further research to be performed so that the translated questionnaire can be further validated among the larger adult population in Malaysia.

## Figures and Tables

**Table 1:** Amendments of the linguistic aspect of the questionnaire

English original phrase	Before amendment	After amendment
Write your score in the box	Tulis markah anda di dalam kotak	Isi markah anda di dalam kotak di bawah
Add up your score	Tambah markah anda	Jumlah markah
Diabetes	Diabetes	Kencing manis
Gestational diabetes	Diabetes ketika mengandung	Kencing manis ketika mengandung
Sister or brother	Saudara perempuan atau lelaki	Adik-beradik
Your weight less than the 1 point column (0 points)	Berat badan anda kurang daripada lajur 1 markah	Berat badan anda kurang daripada berat pada lajur 1 markah (0 markah)
A condition that precedes type 2 diabetes in which blood glucose levels are higher than normal	Suatu keadaan yang mendahului/datang sebelum kencing manis jenis 2 di mana tahap gula dalam darah lebih tinggi daripada biasa	Pradiabetes merupakan suatu keadaan di mana tahap gula dalam darah lebih tinggi daripada biasa tetapi tidak terlalu tinggi untuk diklasifikasikan sebagai kencing manis

**Table 2:** Content validity index

Item	I-CVI	S-CVI/Ave
Q1	1.0	0.93
Q2	1.0	
Q3	1.0	
Q4	1.0	
Q5	1.0	
Q6	0.5	
Q7	1.0	

Note: I-CVI represents item content validity index. S-CVI/Ave represents average of I-CVI scores across all items

**Table 3:** Characteristics of the study participants (*n* = 35)

Characteristics	Frequency (%)	Mean (SD)
Age		20.63 (2.80)
BMI		30.45 (5.99)
Gender		
Male	15 (42.9)	
Female	20 (57.1)	
Ethnicity		
Malay	35 (100.0)	
Chinese	0 (0.0)	
Indian	0 (0.0)	
Other	0 (0.0)	
Marital status		
Married	2 (5.7)	
Single	33 (94.3)	
Divorce	0 (0.0)	
Widow	0 (0.0)	
Education level		
None	0 (0.0)	
Primary school	0 (0.0)	
Secondary school	0 (0.0)	
College/University	35 (100.0)	
Household income		
< RM1,500	28 (80.0)	
RM1,500–RM3,500	5 (14.3)	
> RM3,500	2 (5.7)	

**Table 4:** Face validity index based on the rating of the understandability of items

Item	I-FVI	S-FVI/Ave
Q1	0.94	0.91
Q2	0.97	
Q3	0.86	
Q4	0.91	
Q5	0.94	
Q6	0.91	
Q7	0.86	

Notes: I-FVI represents item face validity index. S-FVI/Ave represents average of I-FVI scores across all items

**Table 5:** Face validity index based on the rating of the clarity of items

Item	I-FVI	S-FVI/Ave
Q1	0.77	0.85
Q2	0.86	
Q3	0.86	
Q4	0.80	
Q5	0.91	
Q6	0.89	
Q7	0.83	

Notes: I-FVI represents item face validity index. S-FVI/Ave represents average of I-FVI scores across all items
